# Assessing Breast Cancer Awareness Among Women in Al Baha, Saudi Arabia: A Cross-Sectional Study Using the Breast Cancer Awareness Measure (BCAM)

**DOI:** 10.3390/medsci13010024

**Published:** 2025-03-01

**Authors:** Mohammad A. Albanghali, Rawan K. Alnemari, Rhaff B. Al Ghamdi, Fatma Alzahraa M. Gomaa, Taif A. Alzahrani, Alya S. Al Ghamdi, Batol M. Albanghali, Yasser M. Kofiah, Eltayeb M. Alhassan, Basim A. Othman

**Affiliations:** 1 Department of Public Health, Faculty of Applied Medical Sciences, Al-Baha University, Al Baha 65779, Saudi Arabia; bothman@bu.edu.sa; 2 Faculty of Pharmacy, Al-Baha University, Al Baha 65779, Saudi Arabia; rawan.alnemari2018@gmail.com (R.K.A.); rahf.9961@gmail.com (R.B.A.G.); ta0yff@gmail.com (T.A.A.); alia0saeed@gmail.com (A.S.A.G.); 3 Department of Pharmacognosy and Medicinal Herbs, Faculty of Pharmacy, Al-Baha University, Al Baha 65779, Saudi Arabia; fgomaa@bu.edu.sa; 4 Faculty of Medicine, Al-Baha University, Al Baha 65779, Saudi Arabia; 445003421@stu.bu.edu.sa; 5 Department of Surgery, Faculty of Medicine, Sciences Al-Baha University, Al Baha 65779, Saudi Arabia; ymkofiah@bu.edu.sa; 6 Department of Oral and Dental Health, Faculty of Applied Medical Sciences, Al-Baha University, Al Baha 65779, Saudi Arabia; emohamed@bu.edu.sa

**Keywords:** breast cancer, BCAM, knowledge, awareness, Saudi

## Abstract

Introduction: Breast cancer (BC) awareness and preventive practices are critical for the early detection and effective management of the disease. This study aimed to assess the level of BC awareness among women residing in Al Baha, Saudi Arabia. Methods: A cross-sectional study was conducted using the Breast Cancer Awareness Measure (BCAM) survey tool to evaluate BC awareness among female residents of Al Baha between June and July 2023. The sample was obtained through the snowball sampling technique. Results: A total of 1007 women participated in the study, with a mean age of 29 ± 10.9 years. Overall awareness of BC warning signs and risk factors was low, with 45% of participants demonstrating poor awareness. Significant positive associations were found between BC awareness and factors such as level of education (*p* = 0.020), employment status (*p* = 0.023), field of study for students (*p* < 0.0001), and average monthly family income (*p* = 0.001). Furthermore, 75% of participants rarely or never practiced breast self-examination, and only 37% of those invited to the Ministry of Health’s screening program had attended. Conclusions: The results highlight a significant lack of awareness and knowledge about BC among women in Al Baha. These findings underscore the urgent need for targeted educational initiatives and awareness campaigns to address this knowledge gap and promote preventive practices.

## 1. Introduction

Breast cancer (BC) is one of the most common types of cancer among women globally, with about 2.3 million new cases diagnosed annually [1,2]. According to the American Cancer Society, one in eight U.S. women will develop BC during their lifetime. Estimates suggest that the number of BC cases worldwide could rise annually to around 3.2 million by 2050 [3]. In emerging countries, the incidence of BC is especially evident due to increased individual life expectancy. Factors including the longer lifespan of women, age, and lifestyle choices all play important roles in increased BC rates [4].

Early cancer detection contributes to timely intervention and treatment, improving patient outcomes and increasing treatment success rates. Advancements in diagnostic methods, such as screening programs and improved imaging technologies, have been crucial in efforts to enhance early detection. Furthermore, increased education and awareness about the symptoms and risk factors of cancer have prompted more people to seek medical attention during the early stages of the disease [5].

BC is a very complicated disease that is influenced by numerous factors, including age, gender, family history, hormonal changes, genetic mutations, obesity, and lifestyle choices [6]. While the incidence of BC is particularly high in Western countries, it is increasingly observed in Asian nations as well, with very high mortality rates [7]. The keys to reducing BC mortality are early detection and treatment, including mammography and clinical breast examinations. In both developing and industrialized nations, a lack of early diagnosis leads to increased mortality. Consequently, enhanced global efforts to detect BC in its early stages are crucial for combatting this disease [8]. In Saudi Arabia, various studies have revealed a significant lack of knowledge among women regarding BC, highlighting the need for early detection [9,10]. For example, Abulkhair colleagues [11] and Radi [12] investigated Saudi women’s knowledge, attitudes, and awareness of BC, concentrating on awareness and breast self-examination. They concluded that there is an urgent need to improve education and awareness about BC to strengthen early detection and prevention. Another study examined BC-related mortality rates in Saudi Arabia, utilizing data from the Saudi Arabian Cancer Registry, in addition to factors that lead to death. The findings showed that understanding these determinants could aid the design of BC interventions seeking to reduce mortality rates [13]. However, several studies have also highlighted the barriers to BC screening, including a lack of knowledge and awareness among both the general population and healthcare workers, reporting that a lack of education, cultural barriers, and inadequate social support could contribute to low screening rates [14,15,16,17].

While previous studies have examined breast cancer awareness in Saudi Arabia, our study specifically focuses on the Al Baha region and provides a detailed breakdown of awareness levels by age group [18,19,20,21,22,23,24,25]. Accordingly, this study aims to assess women’s knowledge and attitudes toward BC in the Al Baha region using the Arabic version of the Breast Cancer Awareness Measure (BCAM) [26].

## 2. Materials and Methods

The cross-sectional study was carried out from 19 June 2023 to 10 July 2023. Stringent ethical considerations were meticulously integrated throughout the study, and explicit informed consent was obtained from all participants.

### 2.1. Study Criteria

The study included Saudi female citizens in Al Baha aged ≥ 18 years who were willing to participate in and were eligible for the study. Relying on the 2010 General Authority for Statistics and Saudi Arabia estimates of the number of females residing in the Al Baha region (N = 179,297), the sample size needed for the study was determined to be 384 with a margin of error of 5% and a confidence interval of 95% [27]. Recruitment was carried out using snowball sampling techniques.

### 2.2. Survey Tool and Measuring Awareness

A self-administered questionnaire was created using a survey tool from a previous study, namely the BCAM [26], which was translated into Arabic with the assistance of a specialist. The Arabic version of the BCAM was then tested for clarity and reliability in a pilot study involving 20 individuals. The final version of the Arabic BCAM incorporated feedback from the pilot study.

The survey tool consists of two sections. The first focuses on socio-demographic information, including questions about participants’ age, marital status, education level, employment status, province of residence, average family monthly income, and the field of study for students. The second section includes a set of 29 items classified into five categories: (i) warning signs (11 items); (ii) risk factors (9 items); (iii) confidence, skills, and behavior (3 items); (iv) delay in seeking medical help (1 item); (v) breast cancer and age (1 item); and (vi) Ministry of Health (MOH) Breast Cancer Screening Program (4 items). It is worth mentioning that due to cultural reasons, one item (risk factors) from the original BCAM (English version) that asked about alcohol consumption was replaced with a question about whether the participant smokes cigarettes. In addition, the questionnaire included a statement for each participant to declare their acceptance of participating in this study. The pooled awareness score was calculated by summing correct responses across four domains: warning signs, risk factors, screening practices, and BC-related age perceptions. Scores ranged from 0 to 42, with higher scores indicating greater awareness. These values reflect participants’ overall understanding of breast cancer, with higher scores correlating to better knowledge of risk factors, symptoms, and the importance of screening practices.

### 2.3. Statistical Analysis

The statistical analysis was carried out using Statistical Package for the Social Sciences^®^ version 20.0 (Armonk, NY, USA: IBM Corp.). Categorical variables were presented as frequencies and percentages, while the distribution of continuous variables was assessed using the mean plus or minus the standard deviation (SD). The relationship between categorical variables was investigated using the chi-square test, while the Kruskal–Wallis test was used to compare medians across various groups of participants, with *p*-values less than 0.05 considered to be statistically significant.

## 3. Results

### 3.1. Participant Characteristics

In this study, 1007 females voluntarily participated by completing an online questionnaire. The majority of the participants, 60%, fell within the age range of 18 to 30 years. Additionally, half of the participants resided in the Al Baha province, and 56% of the participants indicated that their highest qualification was a secondary school certificate. Nearly half of the sample population reported being single. A noteworthy finding was that a large proportion of the participants had a monthly income ranging from 11,000 to 25,000 Saudi Arabian Rials (see Table 1 for detailed information). Moreover, approximately half of the participants (504 individuals) were enrolled in undergraduate programs at the time the survey was conducted, of whom 31% were pursuing degrees in health sciences, 50% in sciences, and 19% in arts.

### 3.2. Awareness of Breast Cancer

#### 3.2.1. Awareness of the Warning Signs and Risk Factors Related to Breast Cancer

The participants identified discharge or bleeding from the nipple and a lump or thickening in the breast as the most well-known warning signs of BC (Table 2). Additionally, over 60% of the participants associated a lump or thickening under the armpit, changes in the size or shape of the breast or nipple, and puckering or dimpling of the breast skin with a higher chance of developing BC. Meanwhile, only 50% of the participants considered pain in the breast or armpits as a sign related to BC. The least known warning signs for BC among the participants were pulling, rashes, or changes in the position of the nipple and redness of the breast skin.

More than 60% of the participants were unaware that being overweight, having children later in life or not at all, starting periods at an early age, experiencing late menopause, and performing less than 30 min of moderate physical activity five times a week were risk factors associated with BC. Conversely, the incidence of previous BC, using hormonal replacement therapy, smoking, and having close relatives with BC were commonly known risk factors associated with BC. Very few participants realized that age is positively associated with risk for BC (Table 3).

#### 3.2.2. Confidence, Skills, and Behavior Related to Breast Cancer

Surprisingly, over 75% of the females in this study rarely or never practiced breast self-examination (BSE). However, 60% of participants reported being confident in their ability to detect changes in their breasts, based on responses categorized as ‘very confident’ and ‘fairly confident’. In addition, only 15% sought advice from doctors for support after experiencing a change in their breasts. More than half of the participants said they would seek help from a specialist within a week if they noticed any changes in their breasts (Table 3). Approximately 70% of the participants confirmed their awareness of the MOH Breast Cancer Screening Program, although only a few demonstrated a deep understanding of it. Of those who received an invitation to attend a breast screening session, around 37% attended.

#### 3.2.3. Pooled Score for Awareness of Breast Cancer

The overall pooled awareness scores ranged from 0 to 41. The mean and standard deviation (SD) were 21 ± 8.02, and the median with interquartile range (IQR) was 21 (16–26). The participants were divided into two distinct groups based on a 50% cut-off of the pooled score. The low-score group, comprising 54% of the participants, had scores ranging from 0 to 21, with a mean and SD of 15.33 ± 4.4 and a median (IQR) of 16 (12–19). The high-score group, representing 46% of participants, had scores between 22 and 42, with a mean and SD of 28.1 ± 5.4 and a median (IQR) of 26 (23–31).

### 3.3. Association Between Sociodemographic Characteristics, Practices, and Awareness of Breast Cancer

**Figure 1:**
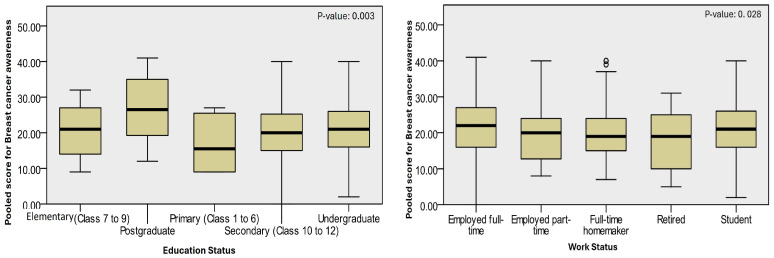

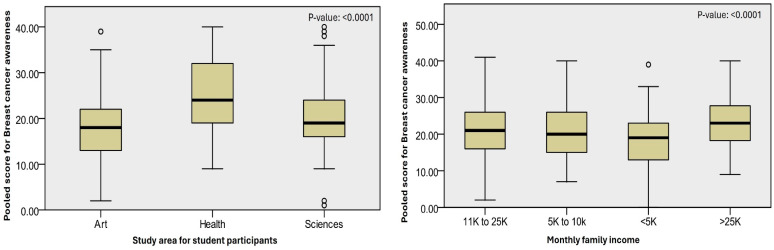
Pooled awareness scores based on various sociodemographic characteristics of participants. ^o^: indicates extreme values. *p*-value estimated using the Kruskal–Wallis test.

The statistical analysis revealed a significant association between the level of education (*p*-value: 0.020), employment status (*p*-value: 0.023), the field of study for students (*p*-value: <0.0001), and average monthly family income (*p*-value: 0.001) with the level of awareness of BC among the participants. This suggests that these factors play a crucial role in determining the level of awareness of BC in the Al Baha female community (see Table 4 and Figure 1). The level of awareness of BC was positively associated with better practices related to BC. This was evident from the increased average awareness scores among participants who regularly practice BSE, are confident about noticing any changes in their breasts, seek advice promptly from specialists when they observe any abnormal changes, and attend breast screenings through the MOH Breast Cancer Screening Program.

## 4. Discussion

This study is the first to estimate BC awareness using the BCAM tool with a large sample of Al Baha women across different age groups, employment statuses, and qualification levels. Understanding individuals’ level of awareness and practices related to BC is vital for the early detection and effective management of the disease. This knowledge is crucial for ensuring timely treatment and improving outcomes, especially for individuals at higher risk of BC. The study has established a baseline level for BC awareness, which can be used as a valuable guide for decision-makers to develop and assess the effectiveness of future BC educational programs aimed at increasing the community awareness of BC. Having sufficient knowledge and awareness about BC and following good practices can improve the likelihood of treatment success for people at risk of developing BC. A woman who is fully aware and able to recognize the early warning signs and symptoms of BC has a greater likelihood of early detection of the disease. This leads to more treatment options, potentially avoiding aggressive procedures, reducing the length of the rehabilitation period, minimizing morbidities, and improving disease-free survival time [28,29]. Several studies have reported that most of the BC cases (47–91%) reported among Saudi women were initially diagnosed at late and advanced disease stages [28,30,31]. This is believed to be a result of various factors, such as a lack of knowledge and awareness, limited access to the healthcare system, and cultural and economic aspects.

Globally, BC is the most common non-communicable disease among females and the main cause of death due to cancer. The literature shows that there is still a lack of knowledge among females about BC [32,33,34,35]. In Saudi Arabia, several studies have found that Saudi females do not differ from other populations in terms of BC awareness [36,37,38,39,40]. Recent studies have reported that awareness of the signs and symptoms of BC ranged between 48% and 71% in cities like Riyadh, Abha, Al Khobar, and Hail [36,37,39]. Meanwhile, Saudi females’ knowledge of risk factors known to be associated with BC has been estimated to be between 47% and 60% [36,37,38,39,40]. Saudi females’ BSE-related knowledge and practices are directly influenced by their awareness of BC warning signs and risk factors. Importantly, studies have indicated that only 27% to 58% of Saudi females were practicing BSE, and 58% had no information on how to perform BSE [41,42]. The estimates regarding BC practices and awareness in the current study are consistent with previous reports in the literature.

Various factors have been shown to influence women’s awareness and behaviors related to BC, including educational attainment, monthly income, family medical history, occupation, and marital status [16,20,42,43]. However, other uncommonly reported and investigated factors might also have an influence, including fear and anxiety, cultural and religious factors, societal stigma, a lack of symptomatic awareness, poor healthcare access and distance, and communication with healthcare providers [44,45,46]. These insights contribute to the growing body of knowledge on cancer prevention and warrant consideration in public health initiatives, awareness campaigns, and policy development. Among Saudi females, it seems that fear of knowing or anxiety about what would happen if one were to be diagnosed with BC are the main reasons that deter women from seeking advice and information and even being involved in a BC educational program [44,47,48]. Of course, any woman would likely experience anxiety and trepidation when contemplating the prospect of being diagnosed with BC. The apprehension surrounding a positive diagnosis can be overpowering. The prospect of undergoing surgical procedures, chemotherapy, or radiation therapy, along with the fear of pain, adverse effects, and lifestyle adjustments associated with treatment, may dissuade women from seeking information or engaging in screening programs. Women who experience anxiety about the disease may avoid conversations or educational materials about BC, thereby impeding their awareness and willingness to adopt preventive measures. Moreover, a fear of embarrassment during clinical breast examinations or mammograms may deter women from seeking such screenings, particularly when compounded by cultural norms and modesty concerns. Some individuals may underestimate their susceptibility to BC, presuming that they are immune to the condition. Anxiety has the potential to distort a woman’s perception of her own risk or can lead to fatalistic thinking, with women believing that their destiny is predetermined. Consequently, they may resign themselves to the inevitability of cancer, resulting in a lack of proactive behavior. Anxiety regarding the physical sensations experienced during mammography or clinical examinations can also discourage women from participating in screening programs [49,50]. Although anxiety and stigma were not directly measured in our study, previous research has consistently identified these as key barriers to BC screening participation [51]. Our findings on low screening uptake align with these broader behavioral influences, suggesting a need for future studies incorporating qualitative measures to better understand their impact in the Al Baha region. Stigma and cultural beliefs remain critical barriers to BC awareness and screening in Al Baha. Social norms, misconceptions about cancer, and fear of diagnosis discourage participation in screening programs. Our findings indicate that women with lower education levels and lower family income were significantly less aware of BC risk factors and screening methods, suggesting an urgent need for targeted interventions. Additionally, younger participants showed higher awareness levels compared to older women, who are at greater risk of BC. This disparity highlights the importance of tailoring awareness programs to different age groups. Furthermore, employed women demonstrated higher BC awareness levels compared to unemployed women, likely due to increased exposure to health-related information in workplaces. Future studies should incorporate qualitative methods to explore how specific sociocultural beliefs shape screening behaviors and compliance with recommended guidelines.

Awareness and practices related to BC can be significantly influenced by stigma and cultural beliefs. In many societies, there is a stigma surrounding discussions about breast health, making it a taboo topic. This can lead to a lack of awareness and silence around the issue. Additionally, certain cultural beliefs attribute BC to ancestral punishment, curses from gods, or the evil eye. These misconceptions can hinder the early detection and treatment of BC. Furthermore, cultural ideals of beauty and body shape can impact how women perceive their breasts and their willingness to seek medical attention. Modesty norms within certain cultures may also affect women’s comfort with BSE and clinical breast examinations, leading to discomfort in discussing or exposing their breasts [49,50]. The current community-based activities conducted for BC Awareness Day in October in Saudi Arabia are insufficient to adequately enhance the knowledge and practices of Saudi women concerning BC. Immediate culturally sensitive interventions are needed to enhance awareness and knowledge within this community. To enhance breast cancer awareness and increase screening participation, we recommend implementing comprehensive educational initiatives, including school-based awareness programs and the integration of women’s health modules into university curricula. Additionally, expanding access to mobile mammography services is crucial for early detection, particularly in remote or underserved areas. Evidence from similar regions demonstrates that community-based outreach programs, hospital-led educational workshops, and digital health campaigns significantly improve breast cancer awareness and screening uptake [52,53,54,55,56]. When tailored to the local sociocultural context, these interventions have proven effective in overcoming screening barriers and promoting early detection [52,53,54,55,56]. In addition to these interventions, recent research indicates that oxidative stress plays a pivotal role in the pathogenesis of breast cancer, with evidence suggesting that antioxidants may mitigate its effects [57]. Studies have demonstrated that dietary and lifestyle interventions aimed at minimizing oxidative damage could serve as potential preventive strategies against breast cancer. Emerging findings emphasize the protective role of antioxidant-rich diets and supplementation in reducing breast cancer risk. These insights contribute to the growing body of knowledge on cancer prevention and warrant consideration in public health initiatives, awareness campaigns, and policy development. To optimize breast cancer prevention efforts in Al Baha, policymakers should consider a multifaceted strategy that engages local communities, healthcare providers, and digital platforms. A structured nationwide program could target different groups of Saudi females based on their age and life stage to ensure effective awareness and prevention measures. Educational programs and awareness events should be incorporated into school curricula to ensure continuous access to accurate breast cancer knowledge for adolescent girls. These initiatives must be culturally appropriate and primarily delivered through school-based activities to foster early awareness. Among young women, universities should introduce a mandatory women’s health module in undergraduate education, covering essential topics such as breast cancer, breastfeeding, personal hygiene, sexual health, maternity, and pregnancy. This structured approach can enhance awareness and promote preventive behaviors among young adults. For women in older age groups, primary health centers should incorporate routine consultations on breast cancer awareness and screening. Healthcare professionals should actively educate women about breast cancer risk factors, self-examination techniques, and available screening services during regular visits. Additionally, home visits should be arranged for disabled women and those facing barriers to healthcare access, ensuring that screening and preventive measures are inclusive and accessible. By implementing these evidence-based strategies, policymakers can ensure that breast cancer awareness efforts reach all segments of the female population, ultimately enhancing early detection rates and reducing breast cancer mortality in Saudi Arabia.

This study is limited by potential sampling bias due to the use of the snowball sampling technique for participant recruitment and data collection. This method may have overrepresented health-conscious individuals, thereby limiting the generalizability of the findings. A more robust approach would involve randomized sampling, which could ensure a more representative sample of the broader community. Additionally, the sample was predominantly composed of younger women, which may reduce the applicability of the findings to older age groups—a population at higher risk of developing breast cancer. Future research should prioritize randomized sampling methods and strive for a more balanced age distribution to enhance the study’s external validity and relevance to high-risk populations.

## 5. Conclusions

The results show that women residing in Al Baha exhibit a limited understanding of and compliance with BC-related knowledge and practices. This observation is consistent with the prevailing situation across the entire nation. It is imperative to implement culturally sensitive interventions to improve awareness and knowledge of BC within the community. This objective can be accomplished by implementing a comprehensive nationwide initiative that focuses on females from different age groups. The primary aim of such a program would be to enhance their knowledge regarding the signs, symptoms, and risk factors of BC, as well as the importance of performing BSE and participating in the MOH’s Breast Cancer Screening Program.

## Figures and Tables

**Table 1:** Sociodemographic characteristics of the participants.

		*N*	%	Age				
				Mean	SD ^†^	Median	IQR ^†^	*p*-Value ^†^
All		1007	100	29.03	10.9	23	20–40	
Age (Year)								
	18–30	601	59.7	20.88	3.2	20	18–22	<0.0001
	31–40	194	19.3	36.32	3.0	36	34–39	
	41–50	190	18.9	44.48	2.6	44	42–46	
	51–60	22	2.2	54.95	3.2	54	52–59	
Education status								
	Primary (Class 1 to 6)	7	0.7	45	9.4	44	39–55	<0.0001
	Elementary (Class 7 to 9)	7	0.7	45	6.97	43	40–52	
	Secondary (Class 10 to 12)	562	55.8	23	8.1	20	18–21	
	Undergraduate	391	38.8	37	7.96	38	31–43	
	Postgraduate	40	4.0	37	6.5	36	33–43	
Provinces								
	Al Baha	502	49.9	27	10.4	21	19–35	<0.0001
	Baljurashi	109	10.8	35	11.6	37	23–44	
	Al Aqeeq	44	4.4	23	6.26	21	19–35	
	Al Mekhwah	143	14.2	34	9.98	32	19–42	
	Al Hajrah	12	1.2	22	3.4	21	20–22	
	Al Mandaq	30	3.0	24	9.58	20	19–23	
	Al Qara	140	13.9	31	10.8	32	19–42	
	Bani Hassan	28	2.7	23	9.28	18	18–29	
Marital status								
	Married	487	48.4	37.5	8.67	39	32–44	<0.0001
	Single	495	49.2	20.22	3.33	20	18–21	
	Divorced	15	1.5	35.87	8.08	40	30–41	
	Widow	10	1.0	42.9	7.94	43	38–48	
Employment status								
	Employed full-time	277	27.5	40.23	6.21	41	37–45	<0.0001
	Employed part-time	50	5.0	31.18	9.42	29	25–41	
	Full-time homemaker	155	15.4	34.9	7.14	34	30–39	
	Retired	21	2.1	52.57	5.79	51	50–59	
	Student	504	50	19.88	1.96	20	18–21	
Study area for student participants								
	Health Sciences	157	31	20	1.38	20	19–20	<0.0001
	Sciences	251	50	20	3.52	19	18–21	
	Art	96	19	21	2.06	20	19–21	
Average family monthly income (SAR) ^†^								
	<5 k	93	9.2	27	9.00	23	20–35	<0.0001
	5 k to 10 k	359	35.7	32	10.5	32	21–40	
	11 k to 25 k	427	42.4	29	11.5	22	19–40	
	>25 k	128	12.7	23	8.34	19	18–22	
Have you, your family, or close friends had cancer?						Yes	No	Don’t Know
	Participant					12 (1)	995 (99)	0 (0)
	Participant’s close family member					191 (19)	783 (78)	33 (3)
	Participant’s other family member					168 (17)	769 (76)	70 (7)
	Participant’s close friends					128 (13)	835 (83)	44 (4)
	Participant’s other friends					180 (18)	754 (75)	73 (7)

^†^ SD: standard deviation, IQR: inter-quartile range, *p*-value estimated using Kruskal–Wallis test, SAR: Saudi Arabian Rials.

**Table 2:** Awareness of warning signs and risk factors of breast cancer.

Items for Examining Awareness of Warning Signs				*N* (%)		
				Yes	No	Don’t Know
1.	Do you think a lump or thickening in your breast could be a sign of breast cancer?			713 (71)	157 (16)	137 (14)
2.	Do you think a lump or thickening under your armpit could be a sign of breast cancer?			659 (65)	179 (18)	169 (17)
3.	Do you think discharge or bleeding from your nipple could be a sign of breast cancer?			764 (76)	100 (10)	143 (14)
4.	Do you think pulling in your nipple could be a sign of breast cancer?			487 (48)	250 (25)	270 (27)
5.	Do you think a change in the position of your nipple could be a sign of breast cancer?			507 (50)	216 (21)	284 (28)
6.	Do you think a nipple rash could be a sign of breast cancer?			532 (53)	257 (26)	218 (22)
7.	Do you think redness of your breast skin could be a sign of breast cancer?			411 (41)	356 (35)	240 (24)
8.	Do you think changes in the size of your breast or nipple could be signs of breast cancer?			601 (60)	214 (21)	192 (19)
9.	Do you think changes in the shape of your breast or nipple could be signs of breast cancer?			615 (61)	194 (19)	198 (20)
10.	Do you think pain in one of your breasts or armpits could be a sign of breast cancer?			530 (53)	299 (30)	178 (18)
11.	Do you think puckering or dimpling of your breast skin could be a sign of breast cancer?			632 (63)	151 (15)	224 (22)
Items for Examining Awareness of Risk Factors		*N* (%)				
		Strongly Agree	Agree	Not Sure	Disagree	Strongly Disagree
1.	Having a history of breast cancer	414 (41)	289 (29)	210 (21)	91 (9)	3 (0.3)
2.	Using HRT (hormone replacement therapy)	260 (26)	306 (30)	370 (37)	62 (6)	9 (1)
3.	Smoking	310 (31)	284 (28)	309 (31)	91 (9)	13 (1)
4.	Being overweight (BMI over 25)	192 (19)	248 (25)	443 (44)	114 (11)	10 (1)
5.	Having a close relative with breast cancer	344 (34)	325 (32)	232 (23)	85 (8)	21 (2)
6.	Having children later in life or not at all	164 (16)	198 (20)	415 (41)	198 (20)	32 (3)
7.	Starting your periods at an early age	116 (12)	136 (14)	404 (40)	280 (28)	71 (7)
8.	Having a late menopause	143 (14)	167 (17)	442 (44)	213 (21)	42 (4)
9.	Doing less than 30 min of moderate physical activity 5 times a week	161 (16)	227 (23)	350 (35)	200 (20)	69 (7)

**Table 3:** Analysis of confidence, skills, behavior, and awareness of the screening program and the association between age and incidence of breast cancer.

			*N*	%	Pooled Awareness Scores		
					Mean ± SD	Median (IQR)	*p*-Value
How often do you check your breasts?							
	At least once a week		25	2.5	23 ± 6.7	23 (18–29)	0.007
	At least once a month		93	9.2	23 ± 7.4	22 (17–28)	
	At least once every 6 months		88	8.7	23 ± 8.4	21 (17–29)	
	Rarely or never		801	79.5	21 ± 8.0	20 (15–25)	
Are you confident you would notice a change in your breasts?							
	Very confident		189	18.8	24 ± 8.9	23 (17–30)	<0.001
	Fairly confident		418	41.5	21 ± 7.3	21 (16–25)	
	Not very confident		312	31	21 ± 7.5	20 (16–26)	
	Not at all confident		88	8.7	17 ± 9.3	16 (10–22)	
Have you ever been to see a doctor about a change you have noticed in one of your breasts?							
	Yes		152	15.1	24 ± 7.5	23 (19–29)	<0.001
	No		495	49.2	21 ± 7.9	20 (15–25)	
	Never noticed a breast change		360	35.7	21 ± 8.1	20 (14–26)	
If you found a change in your breast, how soon would you contact your doctor?							
	Within a week		642	63.8	22 ± 7.6	21 (16–26)	0.051
	Within a month		224	22.2	21 ± 8.5	20 (14–26)	
	More than 4 weeks		141	14	20 ± 8.8	19 (14–26)	
In the next year, who is most likely to develop breast cancer?							
	A 30-year-old woman		195	19.4	-		
	A 50-year-old woman		271	26.9			
	A 70-year-old woman		23	2.3			
	A woman of any age		518	51.4			
Is there an MOH Breast Screening Programme? ^†^							
	Yes		718	71.3	-		
	No		49	4.9			
	Don’t know		240	23.8			
If Yes: At what age are women first invited to the MOH Breast Screening Programme? ^†^							
		Correct	228	35.3	-		
		Not Correct	417	64.7			
If Yes: At what age are women last invited for breast cancer screening?							
		Correct	55	8.5	-		
		Not Correct	590	91.5			
Have you ever been invited for breast screening on the MOH Breast Screening Programme? ^†^							
	Yes		176	24.5	24 ± 7.5	23 (19–29)	<0.001
	No		542	75.5	22 ± 7.9	21 (17–26)	
Have you ever had breast screening on the MOH Breast Screening Programme? ^†^							
	Yes		110	15.3	24 ± 7.4	23 (19–29)	0.037
	No		608	84.7	22 ± 7.9	22 (17–26)	

^†^ MOH: Ministry of Health. -; indicates that this item was included in the calculation of the pooled awareness scores.

**Table 4:** Association between sociodemographic factors and pooled awareness breast cancer scores.

	*N*	%	Pooled Awareness Scores			*p*-Value ^†^		*N*	%	Pooled Awareness Scores			*p*-Value ^†^
			Mean ± SD	Low (0–21)	High (22–42)					Mean ± SD	Low (0–21)	High (22–42)	
All	1007	100	21.2 ± 8.02	546 (54%)	461 (46%)		Marital status						
Age							Married	487	48.4	20.6 ± 7.83	7 (1%)	8 (1%)	0.935
18–30	601	59.7	21.1 ± 8.14	343 (63%)	258 (56%)	0.160	Single	495	49.2	21.7 ± 8.18	265 (49%)	222 (48%)	
31–40	194	19.3	21.4 ± 7.63	98 (18%)	96 (21%)		Divorced	15	1.5	20 ± 6.75	269 (49%)	226 (49%)	
41–50	190	18.9	21.5 ± 6.83	93 (17%)	97 (21%)		Widow	10	1.0	24.8 ± 8.57	5 (1%)	5 (1%)	
51–60	22	2.2	19.6 ± 6.82	12 (2%)	10 (2%)		Employment status						
Education status							Employed full-time	277	27.5	21.9 ± 8.0	131 (24%)	146 (32%)	0.023
Primary (Class 1 to 6)	7	0.7	16.8 ± 8.21	4 (1%)	2 (1%)	0.020	Employed part-time	50	5.0	20.88 ± 9.25	27 (5%)	23 (5%)	
Elementary (Class 7 to 9)	7	0.7	20.85 ± 8.3	4 (1%)	3 (1%)		Full-time homemaker	155	15.4	19.96 ± 7.31	99 (18%)	56 (12%)	
Secondary (Class 10 to 12)	562	55.8	20.93 ± 8.18	319 (58%)	243 (53%)		Retired	21	2.1	18.47 ± 8.15	12 (2%)	9 (2%)	
Undergraduate	391	38.8	21.03 ± 7.49	207 (38%)	185 (40%)		Student	504	50	21.27 ± 8.06	277 (51%)	227 (49%)	
Postgraduate	40	4.0	26.53 ± 8.97	12 (2%)	28 (6%)		Study area for student participants						
Provinces							Health Sciences	157	31	25.28 ± 8.46	58 (21%)	99 (44%)	<0.0001
Al Baha	502	49.9	21.26 ± 8.54	261 (48%)	241 (52%)	0.055	Sciences	251	50	19.97 ± 7.13	149 (54%)	102 (45%)	
Baljurashi	109	10.8	21.94 ± 6.36	57 (10%)	52 (11%)		Art	96	19	18.17 ± 7.13	70 (25%)	26 (12%)	
Al Aqeeq	44	4.4	24.02 ± 7.08	18 (3%)	26 (6%)		Average family monthly income (SAR) ^†^						
Al Mekhwah	143	14.2	20.83 ± 8.55	77 (14%)	66 (14%)		<5 k	93	9.2	18.8 ± 8.09	62 (11%)	31 (7%)	0.001
Al Hajrah	12	1.2	22.58 ± 12.06	7 (1%)	5 (1%)		5 k to 10 k	359	35.7	20.6 ± 7.61	202 (37%)	157 (34%)	
Al Mandaq	30	3.0	21.37 ± 6.60	16 (3%)	14 (3%)		11 k to 25 k	427	42.4	21.4 ± 8.15	230 (42%)	197 (43%)	
Al Qara	140	13.9	19.63 ± 6.75	91 (17%)	8 (2%)		>25 k	128	12.7	23.7 ± 7.95	52 (10%)	76 (17%)	
Bani Hassan	28	2.7	20.59 ± 6.77	19 (4%)	8 (2%)								

^†^*p*-value estimated using chi-square test.

## Data Availability

The raw data supporting the conclusions of this article will be made available by the authors on request.

## Abbreviations

The following abbreviations are used in this manuscript:

BC	Breast cancer
BCAM	Breast Cancer Awareness Measure
MOH	Ministry of Health
BSE	Breast self-examination
IQR	Interquartile range
SD	Standard deviation
